# Sulfated Alginate Reduces Pericapsular Fibrotic Overgrowth on Encapsulated cGMP-Compliant hPSC-Hepatocytes in Mice

**DOI:** 10.3389/fbioe.2021.816542

**Published:** 2022-03-03

**Authors:** Adam M. Syanda, Vera I. Kringstad, Samuel J. I. Blackford, Joachim S. Kjesbu, Soon Seng Ng, Liang Ma, Fang Xiao, Abba E. Coron, Anne Mari A. Rokstad, Sunil Modi, S. Tamir Rashid, Berit Løkensgard Strand

**Affiliations:** ^1^ Department of Metabolism, Digestion and Reproduction, Imperial College London (ICL), London, United Kingdom; ^2^ Department of Biotechnology and Food Science, Norwegian University of Science and Technology (NTNU), Trondheim, Norway; ^3^ Department of Clinical and Molecular Medicine, Norwegian University of Science and Technology (NTNU), Trondheim, Norway

**Keywords:** sulfated alginate, cGMP, hPSC, pluripotent stem cell-derived hepatocytes, PFO, immunogenicity, acute liver failure, immunoisolation

## Abstract

Intra-peritoneal placement of alginate encapsulated human induced pluripotent stem cell-derived hepatocytes (hPSC-Heps) represents a potential new bridging therapy for acute liver failure. One of the rate-limiting steps that needs to be overcome to make such a procedure more efficacious and safer is to reduce the accumulation of fibrotic tissue around the encapsulated cells to allow the free passage of relevant molecules in and out for metabolism. Novel chemical compositions of alginate afford the possibility of achieving this aim. We accordingly used sulfated alginate and demonstrated that this material reduced fibrotic overgrowth whilst not impeding the process of encapsulation nor cell function. Cumulatively, this suggests sulfated alginate could be a more suitable material to encapsulate hPSC-hepatocyte prior to human use.

## Introduction

The liver is one of the most important organs in the body providing numerous vital physiological functions, including protein synthesis, drug metabolism and detoxification, and macronutrient processing (Worman, 1995). Given the significance of the organ in maintaining body homeostasis and functions, its pathology can have detrimental and often fatal effects (Worman, 1995). Furthermore, liver disease is one of the most prevalent causes of mortality globally (Asrani et al., 2019). Currently, the only curative therapy for acute liver failure (ALF) and end-stage liver disease (ESLD) patients is orthotopic liver transplantation (Asrani et al., 2019). Unfortunately, the pool of eligible organ donors is often very limited, which raises the mortality rates of patients affected by ALF that await an HLA-compatible donor (Bernal et al., 2009).

Progress has been made in the development of alternative approaches to the treatment of ALF, including allogeneic hepatocyte transplantation (Dhawan et al., 2010). Despite that this mode of treatment does not require an entire donor liver, there are still some limitations such as obtaining optimal quality of hepatocytes from the source and donor suitability.

Another alternative approach to the treatment of ALF developed in recent years is generation of hepatocytes from pluripotent stem cells (PSCs) (Rashid et al., 2010). Differentiation of PSC into functional hepatocytes holds vast therapeutic potential, the cells are validated for use in the clinical setting and can be tolerated by the patient. Our group has previously demonstrated generation of hepatocytes from current good manufacturing practice (cGMP)-compliant PSC lines (Blackford et al., 2019). These lines were evaluated and shown to be capable of albumin synthesis and CYP3A4 activity, characteristic of the hepatocyte phenotype.

Despite the progress in the development of these cGMP-compliant hepatocytes and their application in *in vitro* studies, the clinical use is still hampered by rejection by the host’s immune cells. To address this issue, a multidisciplinary approach can be implemented to develop a cloaking biomaterial that: can preserve a 3D mass of PSC-Heps, does not elicit an immune response, isolates the transplanted cells from the host’s immune system, and allows pore size-specific flow of molecules essential to metabolism and recovery from liver failure.

Alginate, a polysaccharide isolated from brown algae, i.e., Laminaria hyperborea, has shown to be a worthy candidate as it fulfills many of the desired attributes for use in the clinic. It is a linear co-polymer of β-D-mannuronic acid (M) and α-L-guluronic acid (G). The biopolymer has been granted FDA approval for use in regenerative medicine due to its biocompatibility and safety; moreover, it can be easily handled and has exceptional potential for further functionalization (Sun and Tan, 2013). In the pre-clinical studies in immunodeficient diabetic mice, it has been shown that stem cell-derived islet-like cells encased in alginate successfully maintained viability and reversed hyperglycemia for 12 weeks (Fukuda et al., 2019). Nevertheless, one of the main limitations in using alginate in the clinical setting is the elicitation of foreign body response (FBR) against the biomaterial at the site of implantation leading to fibrotic overgrowth around the microbeads (Bochenek et al., 2018). The deposition of the cellular network and the extracellular matrix, also known as pericapsular fibrotic overgrowth (PFO), can dramatically decrease the diffusion rate of cell products, gases, and nutrients into and out of the microbead compromising the viability and function of the implanted cells (Strand et al., 2017).

Different modifications of alginate-based microbeads have been suggested for improved outcomes in transplantation, e.g., co-encapsulation of drugs and modification of the microsphere structure using other polymer systems and synthetic co-polymers (Orive et al., 2003; Bünger et al., 2005; Ricci et al., 2005; Calafiore et al., 2006; Spasojevic et al., 2014; Vaithilingam et al., 2014; Chen et al., 2015; Hillberg et al., 2015; Park et al., 2017). However, recent modifications to the alginate itself, using alginate beads without a polycation layer, have been demonstrated to reduce the extent and prevalence of PFO on implanted alginate microbeads (Bochenek et al., 2018; Liu et al., 2019). Triazole containing modifications of alginate have been shown to reduce the PFO in both rodents and non-human primates (Bochenek et al., 2018); zwitterion grafting of alginate has also shown a reduction of PFO in mice (Liu et al., 2019). Recently, we have shown that sulfation of alginate reduced the PFO on alginate microbeads in the highly responsive, immunocompetent C57Bl/6J mice (Coron et al., 2022). Previous studies suggest that alginates modified with sulfate groups have anti-inflammatory properties, suppressing pro-catabolic and pro-inflammatory responses from surrounding tissues (Arlov et al., 2016; Arlov et al., 2017). Furthermore, sulfated alginate also shares structural similarities with heparin, a highly sulfated glycosaminoglycan (GAG), and is similarly shown to interact with a range of membrane proteins and growth factors such as bFGF (basic fibroblast growth factor) and HGF (hepatocyte growth factor) (Arlov et al., 2014).

Here, we show that sulfated alginate protects encapsulated cGMP-grade hPSC-derived hepatocytes from an excessive PFO response in the mouse peritoneal cavity, thereby allowing for extended functionality and viability of the encapsulated hepatocyte-like cells in the *in vivo* environment.

## Materials and Methods

### Preparation of Sulfated Alginate

Sulfated alginate was produced according to a previously established method (Arlov et al., 2014). Alginate PRONOVATM UP-MVG, 67% G, 235 kDa (DuPont Nutrition Norge AS d/b/a NovaMatrix, Sandvika, Norway) was dissolved in formamide (Merck KGaA, Darmstadt, Germany) at a concentration of 2.5% (w/v). Chlorosulfonic acid (HClSO3; 99%; Sigma-Aldrich, St. Louis, MO, United States) was added to a final concentration of 2.91% (v/v). The solution was placed in a shaking water bath for 2.5 h at 60°C. The sulfated alginate was precipitated with acetone and re-dissolved in deionized filtered water. The solution’s pH was adjusted to 7 with NaOH and dialyzed (12–14 kDa MWCO, Medicell International Ltd, London, GB) in 0.1 M NaCl, and successive shifts in MQ water until conductivity reached <2 μS/cm. The sulfated alginate was purified using a graphene filter (Millistak+ ® CR40 MCR4023CL3) for 24 h in circular flow with sterile water and freeze-dried until use in the encapsulation process.

### Characterization of Sulfated Alginate

The sulfur content was measured by high-resolution inductively coupled plasma mass spectroscopy at the NTNU Department of Chemistry, Trondheim, Norway, and the degree of sulfation was calculated as per a previously described method (24). Molecular weight averages and dispersity were determined with size exclusion chromatography with multi-angle laser light scattering (SEC-MALLS). Samples were solved to 1 mg/ml in 0.15 M Na_2_NO_3_ and 0.01 M EDTA at pH 6. SEC-MALLS was performed using a TSK G-6000 PW column (Tosoh Bioscience LLC, PA, United States), an LS detector, WTC Dawn Heleos (Wyatt Technology, CA, United States) and an RI detector, Sdodex RI-501 (Showa Denko, Tokyo, Japan). Chromatograms were analyzed in Astra 7.1 (Wyatt Technology, CA, United States). Refractive index increment (dn/dc) was set to 0.15 and 0.13 for alginate and sulfated alginate, respectively, as previously described (Arlov et al., 2017).

### Preparation of Alginate Solutions and Gelling Buffer

This study used alginate (PRONOVA_TM_ UP LVG, 68% G, 237 kDa) as a non-modified alginate control. The sulfated alginate was prepared as per the protocol detailed above. Both alginates were dissolved in 0.3M D-mannitol solutions to maintain optimal solution tonicity and stored overnight at 4°C prior to use. Gelling solution was prepared with 50 mM CaCl_2_, 1 mM BaCl_2_, 10 mM HEPES and 0.15 mM D-mannitol in deionized water. All solutions were pH adjusted to pH 7 and sterile filtrated.

### hPSC-Hep Spheroid Generation

A cGMP-grade human embryonic stem cell line KCL037 (gifted by D. Ilic, King’s College London) was used in the *in vitro* characterization of encapsulated hPSC-Heps. A cGMP-grade induced pluripotent stem cell line CGT-RCiB-10 (gifted by Cell and Gene Therapy Catapult and Dr. Ricardo Baptista) was used in the *in vivo* studies. The line was recovered from a cryopreserved state and thawed as per supplier recommendations. The stem cell line was maintained on Vitronectin XF (STEMCELL Technologies, Vancouver, BC, Canada) coated Corning Costar TC-treated 6-well plates (Sigma–Aldrich, St. Louis, MO, United States) in Essential 8 medium (ThermoFisher Scientific). Hepatocyte-like cells were generated using the previously described differentiation protocol (Blackford et al., 2019). After 20 days of hepatic differentiation, the hPSC-Heps were detached from the culture plates and aggregated into 100–200 μm 800-cell spheroids using AggreWell™400 (STEMCELL Technologies) as per the manufacturer’s recommendations. The aggregated cells were incubated for 2–4 days to ensure stable spheroid formation.

### Encapsulation of hPSC-Hep Spheroids

The hPSC-Hep spheroids were washed and resuspended in 2.0% (w/v) UP-LVG alginate or 2.0% mix of sulfated alginate and UP-LVG (40%; 40:60 ratio) to a concentration of 6.25 × 10^4^ clusters/mL, equivalent to 5 × 10^7^ cells/mL in a final alginate concentration of 1.8%. The microbeads were produced using an electrostatic bead generator (Nisco Engineering) which expelled the spheroid-alginate mixture with a syringe pump at a flow rate of 8 ml/h. An electrostatic ring at 5.3 kV was used to pull the droplets off the nozzle (ID: 0.4 mm, OD: 0.7 mm) reducing the final microbead size to a diameter below 700 μm. The gelling of alginate-cell mix occurred in the dish below the nozzle that contained the gelling solution. Subsequently, the microbeads were washed with 1x Hank’s Balanced Salt solution (containing Ca^2+^) and transferred to CMRL-1066 medium or HepatoZYME-SFM prior to animal surgery or *in vitro* characterization, respectively.

### *In vitro* Functionality Assays

The encapsulated hPSC-Heps were characterized by albumin secretion and ureagenesis to ensure that the encapsulation process did not abolish hepatocyte functions. Albumin secretion was measured by an Enzyme-linked immunosorbent assay using a commercially available kit (Bethyl Laboratories) (*n* = 5). Media from encapsulated cells were collected at 5 and 14 days post-encapsulation. Ureagenesis was measured by a commercially available colorimetric assay kit (QuantiChrom). The encapsulated cells were challenged by 4 mM NH_4_Cl in the culture medium for 24 h, and media supernatant samples were collected and analyzed for urea content subsequently (*n* = 3).

### Permeability Assay

The permeability of sulfated alginate was characterized by the extent of diffusion of FITC probes conjugated to dextran samples with defined molecular sizes. Samples of alginate microbeads were treated with 50 μg/ml solution of FITC-dextran in a range of molecular sizes ranging from 4 kDa to 2 MDa. The samples were incubated for 1 h, 15 and 24 h, and were imaged by Operetta CLSTM High-Content Analysis System (Perkin Elmer) using ×6 magnification and 488 nm excitation laser. The z-height of the plane was selected manually to obtain images penetrating the center of the microbeads. Permeability was measured semi-quantitively in ImageJ (Schneider et al., 2012) by comparing the mean pixel intensity values from the defined central point of each microbead to the mean pixel intensity values of the background (microbead-free area) (n ≥ 11 per group).

### Size Distribution Analysis and Bead Morphometry

Images of the microbeads for analysis were collected using brightfield imaging by Operetta CLS High-Content Analysis System (Perkin Elmer) at ×5 magnification. A Python-based image analysis pipeline was developed to detect the microbeads in the input image using Hough Transform (Duda and Hart, 1972), measure the diameter of each identified microbead and, if present, count the number of hPSC-Hep spheroids within. The program’s output was the number, relative location and the diameter of each microbead, and this data was used for size distribution analysis. The proportion of malformed beads was quantified manually by the researchers.

### Implantation of Encapsulated hPSC-Hep Spheroids

Microencapsulated hPSC-Hep spheroids were washed in PBS and briefly stored in CMRL-1066 serum-free medium prior to implantation. Implantation of alginate encapsulated cells and empty alginate beads was performed as previously described (King et al., 2001; Bochenek et al., 2018). The intraperitoneal space was selected for the implantation site due to proximity to the liver and easy retrieval of the microbeads by saline lavage upon completion of the experiments. The implantation of microencapsulated hPSC-Hep spheroids was performed on 16 immunocompetent C57BL/6J male mice aged 16 weeks; empty alginate microbeads were transplanted into 8 mice as a control. The microbeads were retrieved from the peritoneal cavity after 10 (*N* = 12) and 20 (*N* = 12) days. The intervention (sulfated alginate) and control (unmodified alginate), and no-cell control groups were matched in size (*n* = 4 each). Images of the microbeads were collected using brightfield imaging by Operetta CLSTM High-Content Analysis System (Perkin Elmer), and the extent of PFO was evaluated manually in ImageJ.

### Statistical Methods

The power calculations to determine the sample sizes for all experiments were performed with an effect size of 0.3 and a power of 80% to determine differences at p ≤ 0.05 using G*Power 3.1.9.5.

Statistical differences in permeability of unmodified and sulfated microbeads were determined by repeated Welch’s *t*-test with a sample size of 11 per group. Differences in *in vitro* albumin synthesis were determined by Welch’s *t*-test and the group sample size of 5 was selected as determined by the power calculations. Differences in *in vitro* ureagenesis were determined by Welch’s *t*-test with a sample size of 3 per group. All values are reported as a mean ± SD.

## Results

### Generation of Sulfated Alginate and Alginate Microbeads

**FIGURE 1:**
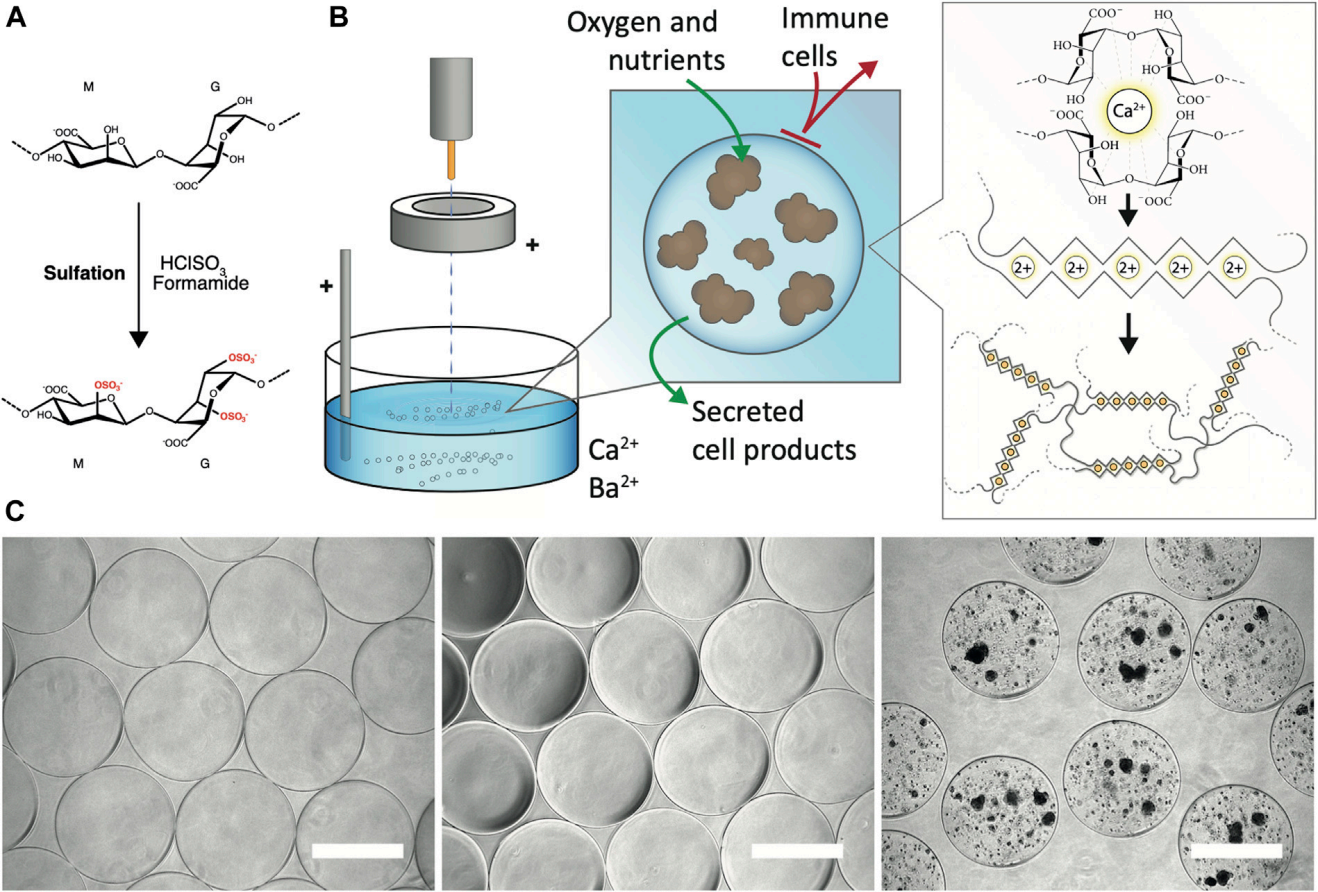
Generation of sulfated alginate and alginate microbeads. **(A)** Sulfation of the alginate by treatment with chlorosulfonic acid in formamide **(B)** Encapsulation of PSC-Hep spheroids. The spheroids in a homogenous mixture of calcium-free alginate solution are pumped out of the encapsulator nozzle. The electrostatic accelerator ring pulls the droplets downwards resulting in a smaller droplet diameter. Subsequently, the cell-alginate droplets are collected in bivalent cation (Ca^2+^ and Ba^2+^; shown as *2+*)-containing solution which promotes crosslinking of predominantly the blocks of L-guluronic acid in the alginate, and generation of solid-phase alginate beads. The alginate matrix allows diffusion of oxygen, nutrients and secreted cell products, and provides a barrier against infiltration of immune cells. **(C)** Gross morphology of alginate microbeads by brightfield microscopy. Unmodified UP-LVG alginate microbeads (left), 40% sulfated alginate microbeads (middle) and hPSC-Heps spheroids encapsulated in 40% sulfated alginate (right) all showed regular morphology. Scale bars: 500 μm.

The sulfation of the alginate has been carried out as per the previously described methodology (Arlov et al., 2017) (Figure 1A). The degree of sulfation per alginate monomer (DS) is dependent on the concentration of chlorosulfonic acid in formamide during the preparation (Arlov et al., 2017). The sulfur content of the product was determined by HR-ICP-MS and the degree of sulfation was further calculated to 0.8 as previously described (Arlov et al., 2017). This implies that approximately 80% of the sugar monomers have substituted hydroxyl with sulfate on C2 or C3 (Figure 1A). After sulfation, the molecular weight (M_W_) was reduced from 235 to 163 kDa. As acidic conditions cause hydrolysis of the glycosidic linkages, a reduction in M_W_ upon sulfation is expected (Arlov et al., 2017). As the sulfation also destroys the specific crosslinking of divalent ions by the blocks of consecutive G-units, the sulfated alginate was mixed with non-modified alginate to maintain the gelling capacity (Arlov et al., 2014). Also, a low concentration of barium ions was used in the gelling solution to stabilize the microbeads (manuscript in review).

The electrostatic encapsulation process (Figure 1B) was optimized to minimize the generation of malformed microbeads and ‘satellites’, residual alginate beads formed by droplet fission due to suboptimal encapsulation parameters. The sulfated alginate is considerably less viscous than the non-modified UP-LVG alginate due to the reduced molecular weight, and thus it is challenging to form regular droplets, resulting in irregular microbead morphology. 40% sulfated alginate showed optimal regularity microbead morphology and an insignificant amount of ‘satellite’ beads after optimization of parameters for encapsulation, resembling the product of pure UP-LVG encapsulation (Figure 1C). The encapsulation of hPSC-Hep in sulfated alginate has shown a successful formation of regular microbeads, which further validated the selected encapsulation parameters (Figure 1C).

### Sulfated Alginate can Be Used to Reliably Encapsulate hPSC-Heps

**FIGURE 2:**
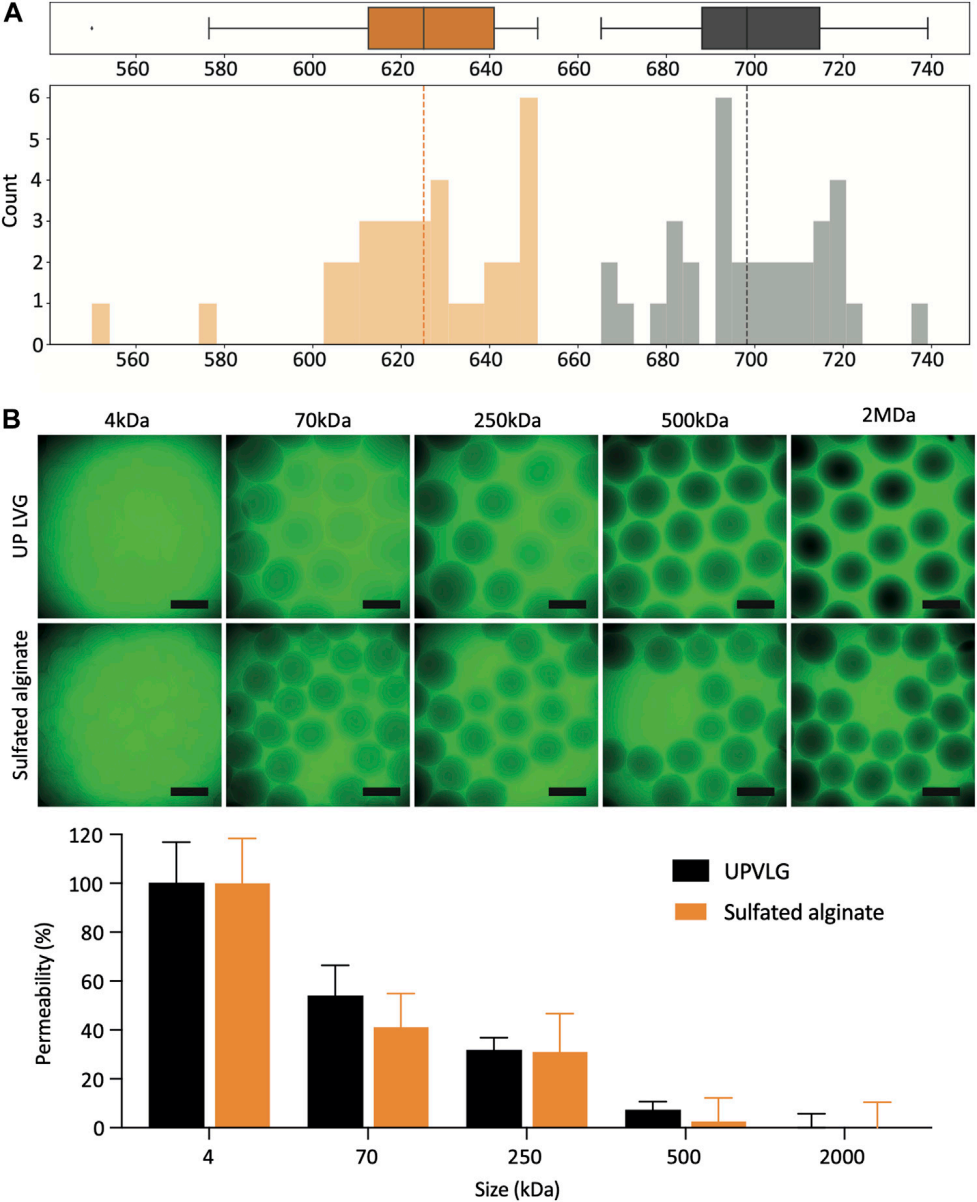
Characterization of sulfated alginate microbeads. **(A)** Size distribution of encapsulated hPSC-Hep spheroids. The boxplots and histograms represent the diameter measurements of unmodified UP-LVG (gray; *n* = 34) and sulfated alginate (orange; *n* = 34). The dashed lines represent the median microbead diameter for each alginate type. **(B)** Permeability of the unmodified UP-LVG and sulfated alginate microbeads (*n* = 11). Selection of representative CLSM images showing different degrees of permeation of FITC-dextran probes at different molecular weights (top). Quantification of FITC-dextran permeation normalized to 4 kDa (100% permeation) and 2 MDa (0% permeation) (bottom). Scale bars: 500 μm. (*p< 0.05).

Upon optimizing the encapsulation parameters and passing the initial qualitative checks, a more in-depth analysis of microbeads was conducted. A Python-based image analysis pipeline determined the microbead diameter, diameter average, and standard distribution (Figure 2A).

Sulfated alginate microbeads were on average smaller than UP-LVG alginate microbeads, with a mean diameter of 624 ± 22 μm and 699 ± 17 μm (mean ± SD), respectively (Figure 2A). The standard deviation of the microbead diameter describes the variability in microbead sizes and ideally should be kept as low as possible. Both alginates showed a comparable standard deviation of diameter, demonstrating that a reproducible encapsulation process across the batch can be maintained for both types of alginate.

The permeability assay was carried out to semi-quantitively compare the differences in permeability of UP-LVG and sulfated alginate (Figure 2B). Permeability of the alginate matrix is a critical parameter in the context of therapy of ALF. Metabolic waste products such as ammonia (17 Da), as well as drugs and their metabolites such as APAP and N-acetyl-*p*-benzoquinone imine (both 0.15 kDa) should be able to diffuse into the microsphere to be cleared by the PSC-hepatocytes. It is also desired that cell products such as albumin (66.5 kDa), Factor V (330 kDa), and urea, the end-product of ammonia metabolism (60 Da), can diffuse out of the microsphere to be utilized or cleared by the therapy recipient. Overall, the permeability of both alginates appeared comparable. There were no significant differences between alginates at 70 and 500 kDa. Although immunoglobulins such as IgG (150 kDa) may travel across the alginate matrix, the decreased permeability above 500 kDa is favorable for immuno-isolation to protect the PSC-hepatocytes from the immune cells and binding to IgM (950 kDa).

### Sulfated Alginate Supports the Functionality of hPSC-Hep Spheroids *in vitro*

**FIGURE 3:**
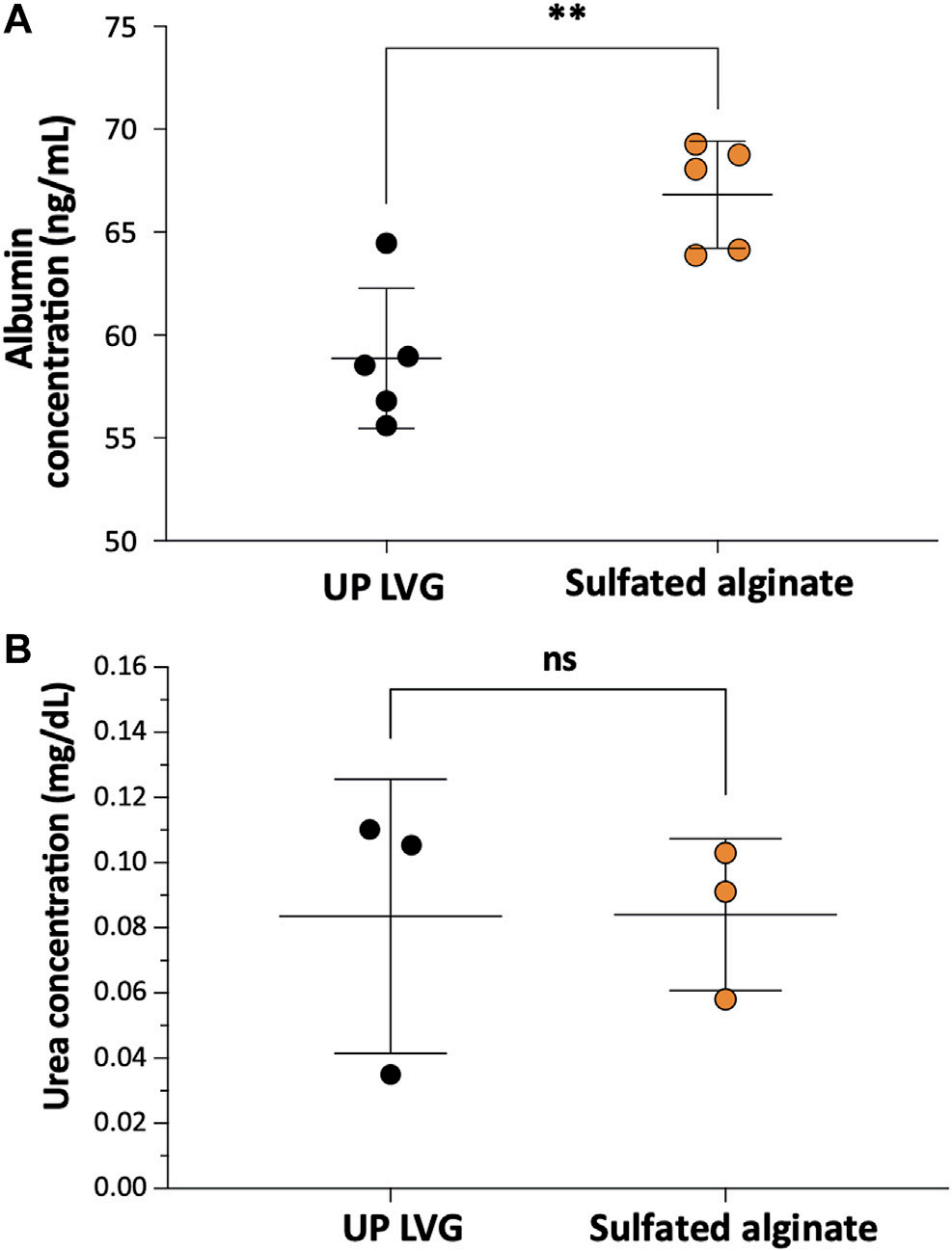
The functionality of encapsulated hPSC-Hep spheroids. **(A)** Comparison of the effects of non-modified and modified alginate microbeads on albumin secretion of encapsulated hPSC-Hep spheroids (*n* = 5). **(B)** Comparison of ureagenesis of hPSC-Hep spheroids encapsulated in UP LVG alginate and sulfated alginate (*n* = 3). (p**<0.01, ns = no significant difference).

Albumin is an essential plasma protein responsible for maintaining the osmotic pressure of the blood and the transport of a range of molecules in the blood. Patients suffering from ALF often present with severe hypoalbuminemia, thus implantation of albumin-synthesizing cells may aid faster recovery. *In vitro* albumin secretion of sulfated alginate-encapsulated hPSC-Heps (66.8 ± 2.6 ng/ml) appeared to be significantly higher (*p* = 0.0032) than in UP-LVG alginate encapsulated hPSC-Heps (58.9 ± 3.4 ng/ml) (Figure 3B). Ammonia is produced by the degradation of amino acids, predominantly by the gut, and it is converted to urea by the liver (Basile and Mullen, 2009). Impaired ammonia metabolism may lead to an increase of blood ammonia to neurotoxic levels. Therefore, the encapsulated hPSC-hepatocytes should be capable of converting ammonia into urea which can be excreted *via* renal excretion. All encapsulated cells were challenged with 4 mM NH_4_Cl and tested for urea production after 24 h of the challenge. The ureagenesis assay for both groups of alginate encapsulation revealed no significant difference (*p* = 0.99) in urea output demonstrating that sulfation of the alginate does not impair ureagenesis of hPSC-Heps (Figure 3B).

### *Ex vivo* assessment of pericapsular fibrotic outgrowth on the non-modified and sulfated alginate microbeads

**FIGURE 4:**
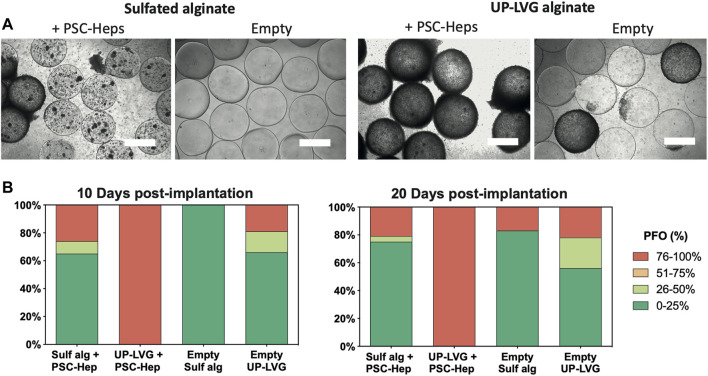
*Ex vivo* assessment of pericapsular fibrotic outgrowth on the non-modified and sulfated alginate microbeads. **(A)** Brightfield microscopy images of alginate microbeads retrieved from mice 10 days after intraperitoneal implantation. hPSC-Heps spheroids encapsulated in sulfated alginate show a lower degree of PFO compared to the spheroids encapsulated in non-modified alginate. Similarly, sulfated alginate empty microbeads show a lower degree of PFO than empty non-modified alginate. Scale bars: 500 μm **(B)** Stack bars showing the average degree of PFO for the alginate microbeads retrieved from the peritoneal cavity of mice at 10 days and 20 days post-implantation timepoints. Upon extraction on day 10 (left), hPSC-Heps encapsulated in sulfated alginate and empty sulfated alginate microbeads showed overall decreased PFO compared to hPSC-Hep-encapsulated and empty UP-LVG alginate microbeads (*n* >= 26 beads retrieved per group); similar but more pronounced difference was observed on day 20 (right) (*n* >= 9 beads retrieved per group).

The encapsulated hPSC-Heps and empty microspheres were implanted into the peritoneal cavity of C57BL/6J mice for a period of 10 and 20 days as this is the most critical time period for the patients suffering from ALF. The treatment in the clinical setting is aimed at the patient’s requiring a “bridging therapy” that will delay the requirement for an orthotopic liver transplant or allow for regeneration of the patient’s own liver. It is, therefore, important to minimize the PFO response within this period. *Ex vivo* assessment of PFO in empty microbeads (Figure 4) after a 10 days-long intraperitoneal implantation showed a higher degree of fibrotic overgrowth (76–100% coverage) in the UP-LVG (19%, *n* = 32) than in sulfated alginate (0%, *n* = 26). Similarly, after 20 days-long implantation, PFO was higher in UP-LVG empty microbeads (22%, *n* = 9) than in sulfated alginate (17%, *n* = 12) microbeads. The lower number of beads on Day 20 was due to a greatly reduced recovery of implanted microbeads compared to Day 10. The difference in PFO was more notable in the encapsulated hPSC-Hep groups. At both implantation timepoints, all UP-LVG-encapsulated-hPSC-Hep microbeads had 100% PFO across all beads (*n* = 27 at day 10, *n* = 37 at day 20). In contrast, in sulfated alginate-encapsulated hPSC-Hep, the high PFO response (76–100% coverage) was found in 26% microbeads on day 10 (*n* = 34) and 21% microbeads on day 20 (*n* = 24) which suggests that sulfated alginate is more suitable for encapsulation of hPSC-hepatocytes in the clinical setting.

## Discussion

Cell encapsulation technology has great potential in the treatment of a range of indications in the clinical setting. Several pre-clinical studies have been undertaken to utilize alginate-based microencapsulation technologies to treat hyperglycemia in mouse (King et al., 2003) and rat models (Chen et al., 2009) of Type 1 diabetes, and also in a few smaller clinical trials (Soon-Shiong et al., 1994; Calafiore et al., 2006; Tuch et al., 2009; Jacobs-Tulleneers-Thevissen et al., 2013). Furthermore, encapsulation of hepatocytes has shown therapeutic efficacy in the treatment of acute liver failure in mouse models of ALF (Mei et al., 2009; Sgroi et al., 2011; Jitraruch et al., 2014), as well as in the clinic for treatment of liver failure in children with urea cycle disorders (Dhawan et al., 2020), in which failure to clear excessive ammonia may result in neurological damage and death. In those cases, the encapsulated allogeneic primary hepatocytes were used to provide short-term support until the native liver regenerates or to support the patient until orthotopic liver transplantation is carried out.

The encapsulation process provides a barrier from the host’s immune cells, provides a scaffold for the embedded cells, and allows a controlled flow of nutrients and cell products. Alginate, a linear polysaccharide from brown seaweed, is a popular biomaterial used to encapsulate cells and bioactive compounds. It is a linear co-polymer of β-D-mannuronic acid (M) and α-L-guluronic acid (G) derived from brown seaweed, i.e., *Laminaria hyperborea*. It forms a stable hydrogel in the presence of divalent cations, such as Ca^2+^, Ba^2+^ and Sr^2+^ (Mørch et al., 2006). Alginate microbeads have been successfully used to encapsulate live cells (Dhawan et al., 2020) and pharmaceutical compounds (Cheng et al., 2018). Successful application of encapsulation technology is dependent on controlled production of optimal microbeads with low variation in size, shape, swelling properties and surface morphology (Lee et al., 2013). In this study, the encapsulation process has been optimized to produce minimal variation in those parameters, as well as to reduce the formation of ‘satellite beads’, residual alginate beads formed from fission of an alginate-cell mix droplet.

The transplantation of non-encapsulated and encapsulated cells into the peritoneal cavity or directly into the liver involves surgical intervention that inherently stimulates tissue repair responses. Normal wound healing processes are associated with a mild postoperative inflammatory response (Cheong et al., 2001). However, due to the presence of foreign bodies such as allogeneic cells or synthetic materials, this response may be exacerbated (Ibrahim et al., 2017). Using alginate for immune isolation of functional xenotransplants has shown to be effective in reducing the immune rejection of the implant (Duvivier-Kali et al., 2004; Qi et al., 2012), and the biomaterial has been shown to have excellent biocompatibility and safety profile (Lee and Mooney, 2012). However, the success rate of this approach, also for allograft transplantation, may be somewhat limited due to the development of a pericapsular fibrotic overgrowth (PFO) over time (Bochenek et al., 2018). PFO reduces the influx of oxygen and nutrients and efflux of waste and functional cell products leading to hypoxia, starvation and apoptosis (Bochenek et al., 2018). Several pre-clinical studies focused on reduction of PFO by modification of the alginate-based microcapsules by coating with polyethylene glycol (Park et al., 2017), CXCL12 (Chen et al., 2015), heparin conjugates (Vaithilingam et al., 2014), methacrylated glycol chitosan (Hillberg et al., 2015) or co-encapsulation with immunomodulatory compounds such as ketoprofen (Ricci et al., 2005). The drawback of some of these modifications is the potential cytotoxicity of the immunomodulatory compound on the encapsulated cells (Ricci et al., 2005). Recent modifications by grafting the alginate itself with triazole or zwitterions have been shown to reduce the PFO on alginate microbeads and maintain the function of encapsulated cells in mice (Bochenek et al., 2018; Liu et al., 2019).

We have recently shown that using sulfated alginates reduces the PFO of implanted empty alginate beads in mice (Coron et al., 2022). Sulfated alginate is also shown to dampen the immune response when used in microbeads and to reduce the inflammatory responses as a soluble polymer in a human whole blood model (Arlov et al., 2016; Arlov et al., 2017). Hence, our approach in this study builds on these previous findings. We attempt to validate this finding *in vivo* in combination with the hPSC-Heps. Sulfated alginate has been shown to interact with local proteins in a manner similar to glycosaminoglycans (GAG) and, in particular, heparin (Arlov et al., 2014) which has previously been shown to have anti-inflammatory effects through inhibition of the complement cascade (Weiler et al., 1978). In conformity with these studies, our findings *in vivo* show that sulfated alginate significantly reduced the prevalence and extent of PFO in both empty and cell-carrying microbeads, thus preserving the movement of oxygen, nutrients and cell products into and out of the microbead. Furthermore, controlled encapsulation of hPSC-Heps is critical in producing cGMP-grade products for use in the clinic. Our experiments showed that encapsulation in sulfated alginate had produced regular microbeads with low variation in size.

Furthermore, sulfated alginate has been shown to bind to many proteins and growth factors, most notably HGF, EGF (epidermal growth factor), bFGF, Interleukin-6 and Platelet-derived growth factor (PDGF) (Freeman et al., 2008). These factors have been associated with promoting hepatocyte proliferation and maintenance of hepatocyte function (Böhm et al., 2010). Although our *in vitro* findings do not show an improvement in ureagenesis in the encapsulated hPSC-Heps, the albumin synthesis, which is correlated with hepatocyte maturation, is significantly upregulated. This demonstrates that sulfated alginate may be beneficial in stimulation and maintenance hepatic functions of encapsulated hPSC-Heps. Further in-depth studies need to be carried out to establish the precise effects of sulfated alginate on hPSC-Hep phenotype maturity, however our findings may readily be translated into the clinical setting after pre-clinical validation.

As demonstrated in our study, sulfated alginate is a suitable candidate for use in the encapsulation of hPSC-Heps in pre-clinical studies. The functionalization of alginate by sulfation enhances albumin synthesis, a function associated with hepatic maturation. Furthermore, sulfated alginate is also beneficial in inhibiting the formation of PFO that blocks the influx and efflux of molecules across the microbead. Future studies will be carried out to fully characterize the specific effects of sulfated alginate on hPSC-Hep maturation and its effects on cell function *in vivo* (e.g. detection of human albumin in mouse serum). These will provide further information on the suitability of this biomaterial in cell therapy applications.

## Data Availability

The raw data supporting the conclusion of this article will be made available by the authors upon reasonable request.
